# Influencing Factors of Nurses’ Well‐Being in Critical Care During Pandemic Era: A Systematic Review

**DOI:** 10.1111/phn.13471

**Published:** 2024-11-12

**Authors:** Maria Emma Musio, Marta Russo, Martina Barbieri, Andrea Moro, Milko Zanini, Loredana Sasso, Annamaria Bagnasco, Gianluca Catania

**Affiliations:** ^1^ Department of Health Sciences University of Genoa Genoa Italy; ^2^ European Institute of Oncology, IRCCS Milan Italy

**Keywords:** burnout, COVID‐19, critical care, intensive care units, nurse, pandemic, well‐being

## Abstract

**Aim:**

To identify factors protecting and hindering the well‐being of critical care nurses during the COVID‐19 pandemic.

**Background:**

The unique work challenges of critical care nurses can undermine their professional and mental well‐being; as evidenced by the prevailing literature on burnout, compassion fatigue, and moral distress. The COVID‐19 pandemic has seen these professionals on the front lines and has raised many questions about professional well‐being. Identifying the factors that protect and hinder the well‐being of critical care nurses would help to develop the strategies necessary to reduce worrying phenomena associated with professional malaise.

**Evaluation:**

A systematic review was conducted using electronic databases including PubMed, CINAHL, Scopus, Cochrane, PsycINFO, and Web on Science.

**Key issues:**

After full text analysis, 25 papers were included in the current systematic review. Factors influencing the well‐being of critical care nurses have been grouped into “Protective” and “Hindering.”

**Conclusions:**

This review shows that critical care nurses’ well‐being is influenced by factors like personal resilience and supportive work environments, which enhance their experience. Challenges include sociodemographic issues and the COVID‐19 pandemic's impact. Addressing these factors is crucial for their well‐being and the effectiveness of healthcare systems.

**Implications for clinical practice:**

The well‐being of nurses is intricately linked to the quality and security of patient care, ultimately influencing clinical outcomes. This review delves deeply into the multifaceted factors that affect the professional well‐being of critical care nurses during the COVID‐19 pandemic. Recognizing these elements is critical for directing health policy toward the development of initiatives that bolster healthcare workers’ welfare. Prioritizing the professional well‐being of nurses is imperative, as it is fundamental in mitigating the increasing inclination towards job turnover, a challenge that is profoundly impacting the healthcare sector.

## 1 Background

Critical care nurses are at high risk of stress due to their daily exposure to suffering patients and caregivers in emotionally intense situations, such as births, illnesses, accidents, and deaths (Alpuente et al. 2018; Molero Jurado et al. 2019; Storti and Visonà 2010). These stressors, combined with the demands for rapid, effective responses to complex and uncertain patient outcomes, make nurses particularly vulnerable to psychological harm, including burnout, compassion fatigue (Epp 2012; Pereira et al. 2016), moral distress (Choe, Kang, and Park 2015), and anxiety disorders, potentially leading to posttraumatic stress and reduced quality of life (Cecere et al. 2023).

International research has primarily focused on the psychological distress experienced by healthcare workers in emergency and intensive care settings (Mäkikangas et al. 2016). However, recent shifts in positive psychology have emphasized well‐being, promoting a balance between personal resources and workplace demands (WHO 2013; Seligman 2011). This perspective highlights the importance of well‐being initiatives for nurses, whose emotional labor is critical to the provision of quality healthcare (Dodge et al. 2012; Guseh, Chen, and Johnson 2015; Parandeh et al. 2022).

In recent years, health policies have increasingly prioritized nurse well‐being, both locally and globally. Mäkikangas et al. (2016) found that most studies on employee well‐being focus on the negative aspects, particularly burnout. This reveals a broader gap in understanding nurse well‐being, especially in intensive care unit (ICU) settings, where the focus remains on distress, fatigue, and burnout (Pereira et al. 2016; Choe, Kang, and Park 2015; Zarei et al. 2016; Burgess et al. 2010).

Two significant global events have exacerbated pressures on nurse well‐being: the 2008 financial crisis and the COVID‐19 pandemic. Both disproportionately impacted critical care nurses and physicians (Thusini 2020; Greenberg et al. 2021). The financial crisis led to widespread healthcare cuts, creating a nursing shortage and unfavorable working conditions (Stuckler et al. 2017). This heightened stress for nurses, resulting in poorer patient care, higher mortality, increased readmission rates, longer hospital stays, and decreased patient satisfaction (Aiken et al. 2014; Lasater et al. 2021).

During the SARS‐CoV‐2 pandemic, healthcare workers, particularly those in first response units, faced extreme stress as they adapted to new living and working conditions. Nurses experienced significant psychological burdens due to factors like the use of personal protective equipment (PPE), departmental reallocation, increased workloads, and the high mortality rate of COVID‐19 patients (Jun, Tucker, and Melnyk 2020; Bambi et al. 2020).

Studies showed that anxiety and fear among healthcare workers increased, driven by challenging working conditions and isolation from family (Hu et al. 2020; Han et al. 2020). Nurses working in COVID‐19 units were especially affected (Hu et al. 2020). ICU nurses, in particular, faced profound psychological impacts from multiple stressors: increased workloads, extended hours, medication shortages, unclear protocols, PPE shortages, fear of infection, family separation, numerous end‐of‐life decisions, and a lack of ICU beds (Heesakkers et al. 2021). Pre‐existing burnout and other psychological conditions worsened during the pandemic, further highlighting the global shortage of staff, especially in intensive care.

Early assessment and intervention for nurses’ psychological needs are crucial. Without proper support, unresolved psychological distress may not only weaken nurses’ immunity, increasing their susceptibility to infection, but also negatively affect the safety and quality of healthcare systems (Baraka, Ramadan, and Hassan 2023).

During the pandemic, nurses also experienced moral distress from exposure to harmful situations, such as suboptimal care, patients dying without relatives, non‐compliance with safety protocols, and working alongside inexperienced ICU colleagues (Andersson et al. 2023; Kok et al. 2021; Silverman et al. 2021). They faced communication breakdowns within ICU teams, resource shortages, substandard care due to limited financial support and time, and persistent staff shortages (Silverman et al. 2021; Kok et al. 2021; Donkers et al. 2021).

Nurses’ emotional well‐being is especially vital in high‐pressure environments like the Emergency Department, where quick decision‐making is essential for optimal patient care (Lateef et al. 2013).

Given these challenges, occupational well‐being has become a pressing priority. There is a growing need to explore strategies that enhance nurses’ professional well‐being. Addressing this issue is also becoming a key political strategy to mitigate the increasing intention to leave the profession and support recruitment and retention efforts.

## 2 Objectives

To identify factors protecting and hindering the well‐being of critical care nurses during the COVID‐19 pandemic.

Specifically, the literature review aims to answer the following research questions:

1. What are the predictors of critical care nurse well‐being in the COVID‐19 era?
2. Which factors hinder the well‐being of the critical care nurse in the COVID‐19 era?

## 3 Methods

### 3.1 Study Design

The present review was developed according to the Joanna Briggs Institute (JBI) guidelines for systematic reviews (Aromataris et al. 2020). A systematic literature review was conducted on the factors that influence the well‐being of critical care nurses in the era of COVID‐19, based on a protocol drawn up according to the recommendations of the JBI Manual for Evidence Synthesis (13, 14), registered in the International Prospective Register of Systematic Reviews (PROSPERO) 2023 registration number CRD42023446542.

To build the research question for this systematic review, we will use the PEOT (Population, Exposure, Outcome, and Type of studies) format was used to identify the significant components of the review's question (Khan et al. 2003).

Population was “Critical care nurses,” “Influencing factors” were identified as the exposure, such as “Well‐being or Burnout” terms were used to indicate the outcome; “COVID‐19 Pandemic era” represented the timing in our research.

Subsequently, we used the Preferred Reporting Items for Systematic Review and Meta‐analyses (PRISMA) guidelines to comprehensively display the identified records’ selection process and report the findings (Moher et al. 2009). The inclusion criteria are described in Table 1.

**TABLE 1:** Inclusion criteria.

Type of participants	Exposure (independent variable)	Outcomes (dependent variable)	Type of studies
All studies, involving: Nurses working in the critical area (ICU and emergency departments)	All studies in which factors that protect or hinder the well‐being of critical care nurses have been identified or assessed with different tools.	The results reported by the authors regarding protective and hindering factors of well‐being were included in the review. The review included studies documenting outcomes on critical care nurses guided by: Personal factors Work‐related environment factors Work experience‐related factors Professional factors Clinical issue‐related factors	All quantitative primary studies on factors influencing intensive care nurse well‐being and the relationships between them were considered eligible. To be included in the review, manuscripts must meet the following inclusion criteria: Primary observational and experimental studies Studies published in English or Italian Description of the favorable and/or hindering factors that influence the nurses’ well‐being Studies conducted in the intensive care setting Studies conducted in the COVID‐19 era. A wide range of study designs was considered appropriate to be as comprehensive as possible and to include the most significant number of studies for this review. Randomized controlled trials (RCTs) Observational studies (e.g., prospective and retrospective cohort studies) Case‐control studies Cross‐sectional analytical studies

Studies meeting the following criteria were excluded:

1. Studies conducted in the pediatric critical care setting.
2. Editorials, case reports, and dissertations.
3. Qualitative studies.

### 3.2 Search Strategies

#### 3.2.1 Electronic Databases

Based on the review question, six databases were searched: PubMed, CINAHL, Scopus, Cochrane, PsycINFO, and Web on Science. Only documents in English and Italian were included. The time limit was set between 2020 and 2023 to limit the review to the period of the COVID 19 pandemic.

Considering the elements of the main research question, a pilot search enabled the identification of keywords consistent with the proposed research questions for the electronic database search (Table 2). The terms included synonyms or specific terms according to each database.

**TABLE 2:** Search concepts and keywords used.

Professional well‐being	Critical care settings	Critical care nurse	Factors	Timing
Well‐being Well‐being Burnout	Critica care Intensive care ICU* Emergency Emergency Medical Service* Emergency Service Hospital Trauma Center* Triage Emergency Department* ICU* Intensive Therapy* Emergency Room ER	Nurse Nurses Nursing staff Nursing personnel Critical care nurse Emergency nurses	Workload Workflow Health workforce Staffing levels Missed care Engagement Moral distress Leadership Nurses practice environment Workplace stress Job satisfaction	COVID era Pandemic era

#### 3.2.2 Keywords

The care services identified by the following terms were considered: “Intensive Care Unit (ICU),” “intensive care,” “emergency department (DEA),” “Emergency Service Hospital,” “Emergency Medical Services,” “Trauma Centers,” “Triage,” “intensive care,” “emergency,” “emergency room (ER).” In case of terminological discrepancy, alternative terms used in international literature were taken into consideration as long as they express a certain correspondence in meaning and a third reviewer was involved in case of disagreement on the terms of the research.

To maximize the identification of potentially relevant manuscripts for inclusion, the bibliographic references of the included articles were selected (reference list scanning) and the sources that cited the included articles were searched in the Scopus database (citation search). All identified bibliographic sources were managed with the EndNote 21 software for Windows (Clarivate Analytics 2021).

### 3.3 Data Extraction

A data extraction sheet was developed according to the JBI guidelines for systematic reviews (Aromataris et al. 2020). The extracted data were reported on a specially structured and previously tested spreadsheet.

Data were extracted independently by at least two researchers. Any discrepancies in the data were resolved by group checking. For each article, the following data were extracted: author, year of publication, country, study design, study population, sample, total sample size, female participants (total) age of participants, study aim(s) treatment intervention(s), outcomes, key findings (protective and hindering factors of well‐being), measurement tools, quality of the study, and limitation of the study.

### 3.4 Data Synthesis

The results of the included studies underwent narrative synthesis, using words and text to summarize and explain the results. Its form varied from a simple account and description of the characteristics of the study, to the context, the quality, and the results. Tables were used to compare the characteristics of the studies and the extracted data.

## 4 Results

### 4.1 Selection of the Studies Included in the Review

**FIGURE 1:**
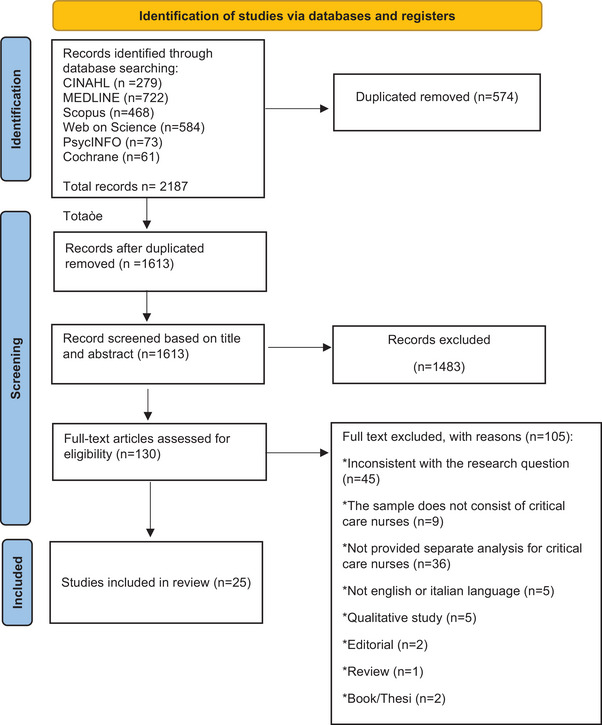
Flow diagram of the literature review process (PRISMA 2009). [Colour figure can be viewed at wileyonlinelibrary.com]

A total of 2187 records were initially identified after searching the databases. Of these articles, 574 were eliminated because they were duplicates. Therefore, 1613 articles were analyzed by title and abstract, and finally, 131 articles underwent full‐text review. After reading the full texts, 25 papers were included in the current systematic review (see Figure 1 and Table 3). As shown in Table 3, all studies were conducted in the hospital setting and professional well‐being was, as usual, not measured directly but by measuring its components such as burnout, anxiety, depression, general health, PTSD, and moral distress. Most of the studies (*n* = 16) involved nurses employed in ICU settings, while a minority of the studies (*n* = 8) included in the review, report findings related to the population of nurses employed in Emergency Department. Only one study analyzes the well‐being of the critical care area nurse both settings (Table 3).

**TABLE 3:** Characteristic of included studies.

Study reference	Country	Department	Sample description	Study aim	Measurement tools used	Outcomes	Quality assessment according JBI guidelines
Blanchard et al. (2022)	USA	Emergency department	701 ED nurses	Evaluate the relationship between the perceived work environment and mental well‐being of a sample of nurses in emergency medical services during the COVID‐19 pandemic	Patient Health Questionnaire for Anxiety and Depression and Job Stress Scale	Anxiety, depression, job stress, perceived work environment	High quality
Smith, Kokoczka, and Cottrell (2023)	USA	ICU	54 ICU nurses	To assess intensive care nurses' perception of the usability of a lounge designed to support them in refreshing and renewing themselves after stressful clinical situation	Apposite developed survey was reviewed by experts for readability, clarity of items, and potential bias.	Nurses' stress level	Medium quality
Hall et al. (2022)	England	ICU	3496 ICU nurses	Describe the prevalence of five mental health in ICU staff over the winter 2020/2021 surge	The 9‐item Patient Health Questionnaire (PHQ‐9); the 6‐item PTSD checklist; AUDIT‐C; the7‐item Generalized Anxiety Disorder (GAD)	Depression, post‐traumatic stress disorder (PTSD), general anxiety disorder and problem drinking	Medium‐high quality
Rhéaume and Breau (2022)	Canada	ICU	236 ICU nurses	Identify factors that are directly and indirectly, associated with burnout and turnover intentions in ICU nurses	Burnout was assessed using the Oldenburg Burnout Inventory (OBI); Moral distress was evaluated using the Modified Moral Distress Scale (MDS‐11)	Burnout, moral distress stress	High quality
McCormick et al. (2023)	Australia	Emergency department	104 ED nurses	Describe the perceptions of occupational stress and coping strategies of ED nurses and doctors and the differences between these two groups	Survey contained 130 questions adapted from 13 validated self‐report measures to assess perceived levels of stress, job demands, burnout, support and recovery experiences in the workplace.	Perceived levels of stress, job demands, burnout, support and recovery experiences in the workplace.	Medium quality
Siam and Alrasheedi (2022)	Saudi Arabian	Emergency department	77 ED nurses	Evaluate the level of burnout among emergency nurses during the COVID‐19 pandemic.	The Copenhagen Burnout Inventory (CBI)	Burnout	Medium quality
Jose, Dhandapani, and Cyriac (2020)	India	Emergency department	120 ED nurses	Determinate burnout and resilience and its associated factors among frontline nurses who provide direct care for the patients in the emergency department	MBI‐HSS (Maslach Burnout Inventory), CD‐RISC (Connor‐ Davinson Resilience Scale‐25)	Burnout and Resilience	Medium quality
Bruyneel et al. (2021)	Belgium	ICU	1135 ICU nurses	Assess the prevalence of burnout risk and identify risk factors among ICU nurses during the first wave of the COVID‐19 pandemic	Maslach Burnout Inventory	Risk of burnout	Medium quality
Heesakkers et al. (2021)	Holland	ICU	726 ICU nurses	Determine the impact of the first COVID‐19 surge on mental well‐being and associated risk factors among ICU nurses	HADS (Hospital Anxiety and Depression Scale); IES‐6, Impact of Event Scale‐6; PTSD, Post‐traumatic stress disorder; NFR (Need For Recovery)	Mental health outcomes (anxiety, depression, and PTSD, NFR)	High quality
Heesakkers et al. (2023)	Holland	ICU	589 ICU nurses	Determine the impact of the second surge of the COVID‐19 pandemic (October 2020 to June 2021) on the mental well‐being of ICU nurses and factors associated with mental health outcomes.	Hospital Anxiety and Depression Scale, Impact of Event Scale‐6, Need For Recovery‐11 questionnaire.	Mental symptoms (anxiety, depression, PTDS); Work‐related fatigue	Medium‐high quality
Pagnucci et al. (2021)	Italy	ICU	245 ICU nurses	Evaluate the effects of the reorganization of an ICU for COVID‐19 patients in the context of the SARS‐CoV‐2 pandemic on well‐being perceived by nurses.	Covid‐19‐NWB (Nurse's well‐being at work scale, in the Italian version)	Nurses well‐being at work	High quality
Şanlıtürk (2021)	Turkey	ICU	262 ICU nurses	Determine the level of occupational stress in intensive care nurses during the COVID‐19 pandemic and factors of perceived stress.	Perceived Stress Scale‐14 (PSS‐14)	Occupational stress, perceived stress	Medium quality
Bruyneel et al. (2023)	Belgium	ICU	2321 ICU nurses	Describe the prevalence of burnout risk and intention to leave the job and nursing profession among ICU nurses and analyze the relationships between these variables and the work environment after two years of the COVID‐19 pandemic	Maslach Burnout Inventory (MBI), The intention to leave the hospital and/or the profession in the next year was assessed dichotomously (yes or no). The Practice Environment Scale of the Nursing Work Index (PES‐ NWI)	Burnout, intention to leave	Medium‐high quality
Yang et al. (2022)	Taiwan	Emergency department	163 ED nurses	Explore the association of the working stress levels with posttraumatic stress disorder symptoms, and burnout as the mediator for this association among emergency nurses during the COVID‐19 pandemic.	Posttraumatic Symptom Scale (PTSS‐10) and Chinese version of the 21‐item Copenhagen Burnout Inventory (CBI).	Occupational burnout and PTSD symptoms	Medium quality
Butera et al. (2021)	Belgium	Emergency department and ICU	T0 = 442 ICU nurses and ED nurses; T1 = 1616 ICU nurses and ED nurses	Assess the prevalence of burnout risk among ICU and emergency nurses before and during the COVID‐19 pandemic and the individual and work‐related associated factors during the pandemic.	Maslach Burnout Inventory (MBI), Job Content Questionnaire	Risk of burnout, social support at work	Medium‐high quality
Vitale et al. (2020)	Italy	ICU	291 ICU nurses	Investigate the burnout syndrome among Italian nurses who engaged in the care of patients with COVID‐19.	Maslach Burnout Inventory (MBI)	Burn out	Medium quality
Ahorsu et al. (2022)	Iran	Emergency department	516 ED nurses	Examine the mediation roles of burnout and job stress in the association between fear of COVID‐19 and mental health among emergency nurses	Fear of COVID‐19 Scale; Occupational Stress Inventory; Maslach Burnout Inventory Human Services Survey for Medical Professionals, fourth edition (MBI‐HSS MP, 4th Ed.), Short Form Health Survey (SF‐12)	Fear of covid, job stress, burnout, mental health	High quality
Baraka et al. (2021)	Egypt	ICU	200 ICU nurses	Identify predictors of stress, anxiety, and depression of critical care nurses in response to COVID‐19 pandemic	Depression, Anxiety and Stress Scale (DASS‐21)	Depression, anxiety, stress:	High quality
Peñacoba et al. (2021)	Spain	ICU	308 ICU nurses	Analyze the moderating effect of personal resources (self‐efficacy, resilience) between stress and the components of physical and mental quality of life in ICU nurses during COVID‐19 pandemic	Depression, anxiety, and stress scale (DASS‐21), General self‐efficacy scale (GSES), resilience scale (RS‐14), and Quality‐of‐life.	Stress, Self‐efficacy, Resilience, and Quality‐of‐life components related to physical and mental health	Medium quality
Kurt Alkan, Taşdemir, and Yıldırım Tank (2022)	Turkey	ICU	116 ICU nurses	Investigate the relationship between the fear of COVID‐19 and depression, anxiety and burnout levels of intensive care nurses	TheFearofCOVID‐19Scale, Depression, Anxiety and Stress Scale Short Form	Fear of COVID‐19, depression, anxiety, stress, burnout	Medium quality
Karadağ and Çiçek (2023)	Turkey	ICU	304 ICU nurses	Determine the depression, anxiety and burnout levels experienced by ICU nurses during the COVID‐19 pandemic and whether there is a relation between these measurements.	Hospital Anxiety and Depression Scale (HADS)	Anxiety, depression, burnout	Medium quality
Damico et al. (2022)	Italy	ICU	375 ICU nurses	Evaluate variations in ICU nurses' mental health status over the COVID‐19 outbreak by quantifying the extent of symptoms of depression, anxiety, and PTSD over time.	Hospital Anxiety and Depression Scale (HADS) PTSD: PCL‐C scale	Anxiety, depression, PTSD	Medium‐low quality
Moreira, Novais, and Martins (2022)	Portugal	Emergency department	60 ED nurses	Evaluate the level of anxiety of nurses in the emergency room about COVID‐19	Hamilton Anxiety Assessment Scale	Anxiety	Medium‐high quality
Vieira et al. (2022)	Brazil	ICU	153 ICU nurses	Study the relationship between burnout dimensions and the work resilience of intensive care nursing professionals in the COVID‐19 pandemic	Maslach Burnout Inventory (MBI), Resilience at work RWS scale Brazil 20, Self‐Reporting Questionnaire (SRQ‐20)	Burnout and resilience	High quality
Alzahrani et al. (2022)	Saudian Arabian	Emergency department	251 ED nurses	Examine the prevalence and influencing factors of hospital anxiety and depression among ED nurses during the COVID‐19 pandemic	Hospital Anxiety and Depression Scale (HADS)	Anxiety and depression	Medium‐high quality

### 4.2 Overview of the Studies Included in the Review

The factors impacting burnout that emerged were thematically categorized and, subsequently divided into protective and hindering factors of critical care nurse well‐being. There were six thematic categories and many sub‐categories identified:

1. Personal factors: divided into (i) Socio‐demographic factors, (ii) Socio‐economic factors, (iii) General health factors (Mental health factors and Physical health factors) and (iv) Other general personal factors.
2. Related to work environment factors: divided into (i) Organizational factors, (ii) Structural architectural factors, (iii) Related to PES‐NWI factors (Nurse manager ability, leadership and support of nurse; Nurse participation in hospital affairs; Collegial nurse‐physician relation; Nurse foundation for quality of care; Staffing and resources adequacy)
3. Work experience‐related factors
4. Professional factors
5. Clinical issue‐related factors
6. COVID‐19 pandemic related factors

In general, it should be noted that far more hindering factors have been identified than protective factors.

### 4.3 Protective Factors

Personal factors are crucial in safeguarding and enhancing the professional well‐being of critical care nurses. Blanchard et al. 2022 study on social‐relational factors during the Covid‐19 pandemic revealed that communication with friends and family significantly supports the well‐being of these nurses.

The concept of “resilience” emerges as a key protective factor for mental health and occupational well‐being in numerous studies, including those by Rhéaume and Breau (2022), McCormick et al. (2023), Peñacoba et al. (2021), Vieira et al. (2022), and Jose, Dhandapani, and Cyriac (2020). Other mental health factors contributing to nurse well‐being, as identified in these studies, include positive personal coping skills, confidence about the future, a sense of accomplishment, and self‐efficacy.

Vieira et al. (2022) pointed out the reciprocal relationship between good sleep quality and occupational well‐being. Alzahrani et al. (2022) noted the benefits of exercise in promoting well‐being.

The influence of the work environment on nurse well‐being is a well‐documented topic in scientific literature. Discussing work environment quality involves considering organizational, structural, and architectural aspects. Hall et al. (2022) identified reduced workload and access to psychological support as key organizational factors for nurse well‐being. Heesakkers et al.’s studies in 2021 and 2023 highlighted the importance of respecting holiday periods and maintaining a work‐life balance. Furthermore, Smith, Kokoczka, and Cottrell (2023) emphasize the presence of a Lavender Lounge in the workplace as a structural‐architectural element that fosters nurse well‐being.

The systematic review underscores various protective factors vital for the professional well‐being of critical care nurses, with a particular emphasis on the work environment as evaluated through the PES‐NWI framework. Numerous studies by different authors have underscored the significant impact of supportive leadership in enhancing and safeguarding the occupational well‐being of nurses. This is evident in the works of Rhéaume and Breau (2022), McCormick et al. (2023), Bruyneel et al. (2023), and Butera et al. (2021). Furthermore, Bruyneel and colleagues’ (2023) study highlights specific protective factors, such as active involvement in hospital affairs and the positive impact of a collegial relationship between nurses and physicians.

In their 2021 research, Pagnucci and colleagues focused on the “Nurse‐physician collegial relationship” aspect of the PES‐NWI sub‐scale. They highlighted several key factors that contribute positively to the well‐being of critical care nurses. These include care and support among nursing peers (also reported in Butera et al. 2021’ study), the liberty to express diverse emotions within the workplace, informal interactions with colleagues, a sense of belonging within the professional community, collaborative spirit and camaraderie among nurses, and the sharing of common objectives along with mutual support.

In their 2023 study, Bruyneel et al. delved into factors influencing the professional well‐being of critical care nurses, particularly those relevant to the PES‐NWI subscale “Nurse foundation for quality of care.” They identified key elements such as exemplary and evidence‐based professional practice, nursing quality care, and the integration of new knowledge, innovations, and improvements as significant contributors to well‐being. Additionally, Pagnucci et al. (2021) highlighted the initial work experience for nurses as a protective factor. In a similar vein, Şanlıtürk (2021) emphasized that increased professional experience plays a protective role in the well‐being of critical care nurses (Table 4).

**TABLE 4:** Critical care nurses’ professional well‐being protective factors.

Critical care nurses’ professional well‐being protective factors				
			Factor	Authors
Personal factors	General Health factors	Mental health factors	Resilience	Rhéaume and Breau (2022)
				McCormick et al. (2023)
				Peñacoba et al. (2021)
				Vieira et al. (2022)
				Jose, Dhandapani, and Cyriac (2020)
			Positive personal coping skills	McCormick et al. (2023)
			More confident about the future	Heesakkers et al. (2023)
			Sense of accomplishment	Ahorsu et al. (2022)
			Self‐ efficacy	Peñacoba et al. (2021)
		Physical health factors	Good sleep quality	Vieira et al. (2022)
			Physical exercise	Alzahrani et al. (2022)
Related to work‐environment factors	Organizational factors		Decreasing workload	Hall et al. (2022)
			Psychological support services for practitioners	Hall et al. (2022)
			Respect holiday periods	Heesakkers et al. (2021)
				Heesakkers et al. (2023)
			Respect work‐life balance	Heesakkers et al. (2023)
	Structural architectural factors		Lavender Lounge	Smith, Kokoczka, and Cottrell (2023)
	Related to PES‐NWI factors	Nurse manager ability, leadership and support of nurse	Supportive leadership	Rhéaume and Breau (2022)
				McCormick et al. (2023)
				Bruyneel et al. (2023)
				Butera et al. (2021)
		Nurse participation in hospital affairs	Participation in hospital affairs	Bruyneel et al. (2023)
		Collegial nurse‐physician relation	Nurse‐physician collegial relation	Bruyneel et al. (2023)
			Assistance and support among nurses	Pagnucci et al. (2021)
				Butera et al. (2021)
			Freedom to express diverse feelings in the work community	Pagnucci et al. (2021)
			Being together with colleagues in an informal way	Pagnucci et al. (2021)
			Sense of belonging to a professional community	Pagnucci et al. (2021)
			Nurses’ togetherness and collaboration	Pagnucci et al. (2021)
			The sharing of common objectives and mutual support	Pagnucci et al. (2021)
		Nurse foundation for quality of care	Exemplary and evidence‐based professional practice	Bruyneel et al. (2023)
			Nursing quality care	Bruyneel et al. (2023)
			New knowledge, innovations and improvements	Bruyneel et al. (2023)
		Staffing and resources adequacy	Structural empowerment of clinical staff	Bruyneel et al. (2023)
Work experience‐related factors			First experience of work for nurses	Pagnucci et al. (2021)
			Increasing in professional experience	Şanlıtürk (2021)
COVID‐19 pandemic‐related factors			Maintain communication with family and/or friends	Blanchard et al. (2022)

Contrary to the findings of the analysis of hindering factors, this review did not reveal protective factors in the professional well‐being of the critical care nurse concerning the following categories: sociodemographic factors, socio‐economic factors; other general personal factors; professional factors and clinical issue‐related factors.

### 4.4 Hindering Factors

In exploring factors that hinder the professional well‐being of critical care nurses, various personal and socio‐demographic elements have been identified in recent research. A study by Hall et al. (2022) and Bruyneel et al. (2023) points to young age as a potential challenge. Conversely, Baraka, Ramadan, and Hassan (2023) suggest that higher age can also be a hindering factor. The aspect of gender is highlighted in multiple studies, with Baraka, Ramadan, and Hassan (2023), D'Amico et al. (2022), Moreira, Novais, and Martins (2022), Alzahrani et al. (2022), and Vitale et al. (2020) noting that being female may impact well‐being negatively.

Marital status is another significant factor, with Baraka, Ramadan, and Hassan (2023) indicating that getting married can pose challenges to nurses' professional well‐being. Age‐specific hindrances are also noted, with Karadağ and Çiçek (2023) finding the 45–55 years age group and D'Amico et al. (2022) the 31–40 years age group as particularly affected. Furthermore, Moreira, Novais, and Martins (2022) identified not having children as a factor that can negatively influence the well‐being of critical care nurses. These findings underscore the complex interplay of personal characteristics in the professional lives of critical care nurses.

In examining socio‐economic factors that adversely affect the professional well‐being of critical care nurses, certain key elements have been identified in recent studies. Blanchard et al. (2022) specifically highlighted two such factors.

Firstly, the concern about their community's response to COVID‐19 emerged as a significant stressor. This worry encompasses not just the immediate health implications but also the broader social and economic impacts of the pandemic, reflecting the nurses' deep engagement with their community's well‐being.

Secondly, financial difficulties were identified as a critical factor. This points to the economic challenges faced by critical care nurses, which can be exacerbated in times of public health crises like the COVID‐19 pandemic. Such financial strains not only affect their personal lives but also have the potential to impact their professional performance and overall well‐being.

These findings from Blanchard et al. (2022) underscore the multifaceted nature of challenges faced by critical care nurses, extending beyond the clinical environment to encompass broader socio‐economic concerns.

In exploring the general health factors that impact the well‐being of critical care nurses, both mental and physical health elements play significant roles, as highlighted in recent research.

Mental health factors are a primary concern, with studies by Hall et al. (2022), Baraka, Ramadan, and Hassan (2023) identifying the presence of mental disorders, such as anxiety, depression, and PTSD, as major hindrances to the well‐being of critical care nurses. These conditions, prevalent in high‐stress healthcare environments, underscore the intense psychological and emotional demands of nursing, particularly in critical care settings.

In terms of physical health factors, Karadağ and Çiçek (2023) bring attention to the impact of chronic diseases on nurses’ professional well‐being. The presence of long‐term health conditions can significantly affect a nurse's ability to perform their duties effectively and can contribute to increased stress and reduced job satisfaction. Additionally, Baraka, Ramadan, and Hassan (2023) highlight the presence of physiological problems, further emphasizing the link between physical health and the ability to maintain a high level of professional performance in demanding healthcare environments.

Another crucial aspect identified by Smith, Kokoczka, and Cottrell (2023) pertains to general personal factors, specifically home and personal issues. These factors, which can range from family responsibilities to personal life challenges, play a significant role in shaping the overall well‐being of critical care nurses. They influence not just the personal lives of these professionals but also have direct implications for their work life and professional satisfaction.

These findings collectively illustrate the multifaceted nature of challenges faced by critical care nurses, spanning mental and physical health, as well as personal life circumstances, all of which crucially impact their professional well‐being.

The work environment of critical care nurses is heavily influenced by various organizational factors, as evidenced in several recent studies. These factors have a substantial impact on the professional well‐being of these healthcare professionals.

A recurring issue identified in multiple studies, including those by McCormick et al. (2023), Şanlıtürk (2021), and Bruyneel et al. (2021), is excessive workload. This challenge is further compounded by time pressure, as noted by Smith, Kokoczka, and Cottrell (2023). Such conditions can lead to burnout and reduced job satisfaction.

Working in an academic hospital, as highlighted by Heesakkers et al. 2021, 2023), presents its own set of challenges, potentially due to the dual demands of clinical care and academic responsibilities. Night shifts, mentioned by Şanlıtürk (2021), also pose unique challenges, impacting nurses’ health and well‐being.

The lack of or limited access to quality material resources, a concern raised by Smith, Kokoczka, and Cottrell (2023), Bruyneel et al. (2021), and Baraka, Ramadan, and Hassan (2023), is another significant factor. This shortage can hinder the ability of nurses to provide optimal care.

Inadequate or unclear rules and procedures, as well as unsafe orders or policies from those in charge, identified by Smith, Kokoczka, and Cottrell (2023), can create a stressful and potentially hazardous work environment. Overcrowding, noted by McCormick et al. (2023), and a generally adverse working environment, as mentioned by Blanchard et al. (2022) and Şanlıtürk (2021), further contribute to the stress experienced by nurses.

The taxing nature of the work environment, characterized by noise, hectic activity, and overcrowding, as described by Smith, Kokoczka, and Cottrell (2023), can be overwhelming. Additionally, Alzahrani (2022) points out the unique challenges of working in urban areas, which may involve dealing with a higher volume of patients and potentially more complex cases.

These findings collectively underscore the impact of organizational and environmental factors on the well‐being of critical care nurses, highlighting the need for supportive and well‐resourced work settings to enhance their professional satisfaction and overall health.

The work environment of critical care nurses is significantly shaped by several factors related to the PES‐NWI (Practice Environment Scale of the Nursing Work Index), as identified in recent research. These factors can either support or hinder the professional well‐being of nurses in critical care settings.

In terms of nurse manager ability, leadership, and support, inadequate supportive leadership has been noted as a key hindrance. Studies by Blanchard et al. (2022), Bruyneel et al. (2023), and Heesakkers et al. (2021) emphasize how the lack of effective leadership can negatively impact the morale and efficiency of nursing staff.

Concerning the collegial nurse‐physician relationship, issues like disagreement or conflict with colleagues, as highlighted by Smith, Kokoczka, and Cottrell (2023), and inadequate collaboration with other healthcare workers, as noted by Heesakkers et al. (2021), can create a challenging work environment. These relational difficulties can lead to increased stress and decreased job satisfaction among nurses.

Regarding the Nurse foundation for quality of care, a critical factor identified is the inability to provide optimal care, as discussed by McCormick et al. (2023). This challenge can stem from various systemic and organizational limitations, impacting the nurses’ sense of accomplishment and professional efficacy.

For staffing and resource adequacy, several studies highlight significant concerns. The number of hours worked per shift, as noted by Heesakkers et al. (2021) and Şanlıtürk (2021), can lead to exhaustion and burnout. Poor skill‐mix, identified by McCormick et al. (2023), and lack or inadequacy of staffing, as mentioned by Smith, Kokoczka, and Cottrell (2023); Heesakkers et al. (2021), and Hall et al. (2022), Şanlıtürk (2021), are also critical issues. These factors can create an overwhelming workload and reduce the quality of patient care, thereby impacting the nurses' sense of professional achievement and satisfaction.

Overall, these findings from various studies underscore the importance of addressing these PES‐NWI related factors in the work environment to enhance the well‐being and professional effectiveness of critical care nurses.

Work experience and educational background are significant factors influencing the well‐being and performance of critical care nurses, as highlighted in various studies.

Reduced professional experience in the ICU has been identified as a factor that can impact the performance of nurses negatively. This is noted in studies by Hall et al. (2022), Şanlıtürk (2021), and Baraka, Ramadan, and Hassan (2023). Conversely, Alzahrani et al. (2022) found that an increase in professional experience can positively influence nursing practice.

Hall et al. (2022) also point out that lower professional grade can be a limiting factor, potentially affecting confidence and decision‐making abilities. In terms of educational attainment, Siam and Alrasheedi (2022) highlight that a lower educational level can hinder a nurse's ability to adapt to complex situations in critical care settings. Similarly, Baraka, Ramadan, and Hassan (2023) emphasize the absence of continual education on infection control as a notable gap, especially in the context of the COVID‐19 pandemic.

Interestingly, a high educational level is also noted as a factor by Bruyneel et al. (2023) and Baraka, Ramadan, and Hassan (2023), suggesting that it might bring its own set of challenges, possibly due to increased expectations or more complex responsibilities.

Work experience duration also plays a role. Karadağ and Çiçek (2023) observed that nurses with 16 years of experience or more, as well as those with less than 1 year, face unique challenges. Additionally, working in an ICU, especially for those who dislike it (Karadağ and Çiçek, 2023), and having 0–5 years of experience (D'Amico et al. 2022) are factors that can impact the well‐being of nurses in different ways.

These studies collectively underscore the nuanced and varied impact of work experience, professional grade, and educational level on the professional well‐being and effectiveness of critical care nurses. They highlight the need for tailored support and development opportunities at different stages of a nurse's career. Professional factors play a significant role in shaping the experiences and well‐being of critical care nurses, as highlighted by various studies.

Meeting the emotional needs of patients and their families is a crucial aspect of nursing care, as noted by Smith, Kokoczka, and Cottrell (2023). This responsibility, while central to nursing, can be emotionally taxing and contribute to stress.

The death of a patient, a reality in critical care settings, is another significant factor that impacts nurses emotionally and professionally, as observed by Smith, Kokoczka, and Cottrell (2023) and McCormick et al. (2023). The emotional toll of such experiences can have lasting effects on nurses' well‐being.

Assault or aggression from a patient's family, identified by Smith, Kokoczka, and Cottrell (2023), poses not only a physical risk but also contributes to psychological stress. Additionally, the complexity of tasks that require a high level of attention, as mentioned by Smith, Kokoczka, and Cottrell (2023), adds to the cognitive load and can lead to fatigue and burnout.

Prolonged intense exposure to professional stressors, noted by Smith, Kokoczka, and Cottrell (2023) and McCormick et al. (2023), is a key factor contributing to chronic stress and burnout among nurses. Moral distress, as identified by Rhéaume and Breau (2022), arises when nurses are unable to act according to their ethical beliefs, further contributing to psychological strain.

The intention to leave the profession, discussed by Rhéaume and Breau (2022), can be a result of cumulative stress and dissatisfaction, while workplace violence, as noted by McCormick et al. (2023), adds to the challenging nature of the nursing profession. Inadequate salary, highlighted by Şanlıtürk (2021) and Baraka, Ramadan, and Hassan (2023), is a practical concern that can affect job satisfaction and the ability to meet personal and family needs, adding to the overall stress experienced by nurses.

These studies collectively underscore the multifaceted professional challenges faced by critical care nurses, encompassing emotional, cognitive, ethical, and practical aspects, all of which significantly influence their professional satisfaction and overall well‐being. The COVID‐19 pandemic has introduced numerous factors that have significantly impacted the work environment and well‐being of critical care nurses, as highlighted in several studies.

A key concern has been the perceived inadequate workplace safety against COVID‐19, identified by Jose, Dhandapani, and Cyriac (2020). This is compounded by exposure to infectious diseases, as noted by McCormick et al. (2023). Working in a COVID‐19 ICU, as mentioned by Heesakkers et al. (2021), brings an additional set of challenges and risks.

The fear of contracting COVID‐19 is a widespread concern among nurses, as evidenced in studies by Heesakkers et al. (2021), Şanlıtürk (2021), Smith, Kokoczka, and Cottrell (2023), Ahorsu et al. (2023), Kurt Alkan, Taşdemir, and Yıldırım Tank (2022), and Yang et al. (2022). This fear extends beyond personal safety to the worry of transmitting the virus to family members, a significant stressor as reported by Smith, Kokoczka, and Cottrell (2023); Heesakkers et al. (2021); Jose, Dhandapani, and Cyriac (2020); and Şanlıtürk (2021).

The lack of PPE has been another major issue, as highlighted by Bruyneel et al. (2021), Şanlıtürk (2021), and Butera et al. (2021), increasing the risk of infection and adding to the anxiety of healthcare workers.

Nurses have also faced the emotional challenge of living apart from their families to minimize the risk of spreading the virus, as discussed by Yang et al. (2022). Additionally, the lack of recognition for their critical role during the pandemic, from the general public, policymakers, and the media, as noted by Bruyneel et al. (2023), has affected morale.

The pandemic has also led to an increased workload for nurses, as identified by Bruyneel et al. (2023) and Butera et al. (2021). This has been exacerbated by the need to employ non‐ICU nurses in ICU settings during peak pandemic waves, as mentioned by Bruyneel et al. (2023). The time spent caring for COVID‐19 patients, as reported by Baraka, Ramadan, and Hassan (2023), and dealing with the infection among colleagues, as noted by Baraka, Ramadan, and Hassan (2023), have further increased the strain on nurses.

Lastly, the challenge of patients not complying with infection control rules, as observed by Yang et al. (2022), adds another layer of complexity and risk in managing patient care during the pandemic. These factors collectively highlight the multifaceted and profound impact of the COVID‐19 pandemic on the work and well‐being of critical care nurses, presenting unique challenges and stressors in their professional environment (Table 5).

**TABLE 5:** Critical care nurses’ professional well‐being hindering factors.

Critical care nurses' professional well‐being hindering factors				
			Factor	Authors
Personal factors	Sociodemographic factors		Young age	Hall et al. (2022)
				Bruyneel et al. (2023)
			Higher age	Baraka, Ramadan, and Hassan (2023)
			Female gender	Baraka, Ramadan, and Hassan (2023)
				D'Amico et al. (2022)
				Moreira, Novais, and Martins (2022)
				Alzahrani et al. (2022)
				Vitale et al. (2020)
				Baraka, Ramadan, and Hassan (2023)
			Getting married	Baraka, Ramadan, and Hassan (2023)
			Age group 45–55 years	Karadağ and Çiçek (2023)
			Age group 31–40 years	D'Amico et al. (2022)
			Don't have children	Moreira, Novais, and Martins (2022)
	Socio‐economic factors		Being worried about their community's response to COVID‐19	Blanchard et al. (2022)
			Financial difficulties	Blanchard et al. (2022)
	General Health factors	Mental health factors	Presence of mental disorders (anxiety, depression, PTSD)	Hall et al. (2022)
				Baraka, Ramadan, and Hassan (2023)
		Physical health factors	Chronic disease	Karadağ and Çiçek (2023)
			Presence of physiological problems	Baraka, Ramadan, and Hassan (2023)
	Other general personal factors		Home/personal issue	Smith, Kokoczka, and Cottrell (2023)
Related to work environment factors	Organizational factors		Excessive workload	McCormick et al. (2023)
				Şanlıtürk (2021)
				Bruyneel et al. (2021)
			Excessive workload/time pressure	Smith, Kokoczka, and Cottrell (2023)
			Academic Hospital	Heesakkers et al. (2021)
				Heesakkers et al. (2023)
			Night shift	Şanlıtürk (2021)
			Lack of/low quality/low accessibility to material resources	Smith, Kokoczka, and Cottrell (2023)
				Bruyneel et al. (2021)
				Baraka, Ramadan, and Hassan (2023)
			Lack of appropriate rules/procedures	Smith, Kokoczka, and Cottrell (2023)
			Unsafe orders/policies from the person in charge	Smith, Kokoczka, and Cottrell (2023)
			Overcrowding	McCormick et al. (2023)
			Adverse working environment	Blanchard et al. (2022)
				Şanlıtürk (2021)
			Taxing work environment (noisy, hectic, crowded)	Smith, Kokoczka, and Cottrell (2023)
			Working in an urban area	Alzahrani (2022)
	Related to PES‐NWI factors	Nurse manager ability, leadership and support of nurse	Inadequate supportive leadership	Blanchard et al. (2022)
				Bruyneel et al. (2023)
				Heesakkers et al.(2021)
		Collegial nurse‐physician relation	Disagreement/conflict with colleagues	Smith, Kokoczka, and Cottrell (2023)
			Inadequate collaboration with other healthcare workers	Heesakkers et al. (2021)
		Nurse foundation for quality of care	Inability to provide optimal care	McCormick et al. (2023)
		Staffing and resources adequacy	Hours worked per shift	Heesakkers et al. (2021)
				Şanlıtürk (2021)
			Poor skill‐mix	McCormick et al. (2023)
			Lack of or inadequate staffing	Smith, Kokoczka, and Cottrell (2023)
				Heesakkers et al. (2021)
				Hall et al. (2022)
				Şanlıtürk (2021)
Work experience related factors			Reduced professional experience in ICU	Hall et al. (2022)
				Şanlıtürk (2021)
				Baraka, Ramadan, and Hassan (2023)
			Increasing in professional experience	Alzahrani et al. (2022)
			Lower professional grade	Hall et al. (2022)
			Low educational level	Siam and Alrasheedi (2022)
			Absence of continual education on infection control	Baraka, Ramadan, and Hassan (2023)
			High educational level	Bruyneel et al. (2023)
				Baraka, Ramadan, and Hassan (2023)
			Working experience for 16 years or more	Karadağ and Çiçek (2023)
			Working experience for less than 1 year	Karadağ and Çiçek (2023)
			Unlike working in ICU	Karadağ and Çiçek (2023)
			Working experience between 0 and 5 years	D'Amico et al. (2022)
Professional factors			Meeting the emotional needs of the patient's family	Smith, Kokoczka, and Cottrell (2023)
			Meeting the emotional needs of the patient	Smith, Kokoczka, and Cottrell (2023)
			Death of a patient	Smith, Kokoczka, and Cottrell (2023)
				McCormick et al. (2023)
			Assault/aggression from the patient's family	Smith, Kokoczka, and Cottrell (2023)
			Task complexity requiring a high level of attention	Smith, Kokoczka, and Cottrell (2023)
			Prolonged intense exposure to professional stressors	Smith, Kokoczka, and Cottrell (2023)
				McCormick et al. (2023)
			Moral distress	Rhéaume and Breau (2022)
			Intention to leave	Rhéaume and Breau (2022)
			Workplace violence	McCormick et al. (2023)
			Inadequate salary	Şanlıtürk (2021)
				Baraka, Ramadan, and Hassan (2023)
Clinical issue‐related factors			Decisional dilemmas or uncertainty regarding patient survival/end‐of‐life care	Smith, Kokoczka, and Cottrell (2023)
			Inappropriate expectations or behaviors for patients' family	Smith, Kokoczka, and Cottrell (2023)
			Inaccurately informed patients	Smith, Kokoczka, and Cottrell (2023)
			Patient's preferences being ignored	Smith, Kokoczka, and Cottrell (2023)
			Disagreement with family regarding the plan of care	Smith, Kokoczka, and Cottrell (2023)
			Disagree with the care team's plan of care	Smith, Kokoczka, and Cottrell (2023)
			Interruption/interference/distraction/unanticipated changes	Smith, Kokoczka, and Cottrell (2023)
			Patients’ family response to patient conditions	Smith, Kokoczka, and Cottrell (2023)
			Unsuitability of care (futility or over/under aggressiveness of therapeutics)	Smith, Kokoczka, and Cottrell (2023)
			High‐acuity patients	McCormick et al. (2023)
			Sexual abuse of a patient	McCormick et al. (2023)
			Worsening patient's clinical conditions	Şanlıtürk (2021)
Covid‐19 pandemic‐related factors			Perceived inadequate workplace safety against COVID‐19	Jose, Dhandapani, and Cyriac (2020)
			Infectious disease exposure	McCormick et al. (2023)
			Working in COVID‐19 ICU	Heesakkers et al. (2021)
			Fear of getting infected by COVID	Heesakkers et al. (2021)
				Şanlıtürk (2021)
				Smith, Kokoczka, and Cottrell (2023)
				Ahorsu et al. (2023)
				Kurt Alkan, Taşdemir, and Yıldırım Tank (2022)
				Yang et al. (2022)
			Lack of protective equipment (DPI)	Bruyneel et al. (2021)
				Şanlıtürk (2021)
				Butera et al. (2021)
			Fear of exposing my family to COVID‐19	Smith, Kokoczka, and Cottrell (2023)
				Heesakkers et al. (2021)
				Jose, Dhandapani, and Cyriac (2020)
				Şanlıtürk (2021)
			Living apart from family	Yang et al. (2022)
			Lack of recognition of their role through the pandemic (by the general public, policymakers, the media)	Bruyneel et al. (2023)
			Lack of social support	Yang et al. (2022)
			Increased workload due to Covid‐19	Bruyneel et al. (2023)
				Butera et al. (2021)
			Compensating excessive workload during pandemic wave employing non ICU nurse in ICU	Bruyneel et al. (2023)
			Time spent on caring Covid patients per week	Baraka, Ramadan, and Hassan (2023)
			Number of colleagues infected by Covid	Baraka, Ramadan, and Hassan (2023)
			Patients not complying with infection control rules	Yang et al. (2022)

Contrary to the findings of the analysis of protective factors, this review did not reveal hindering factors in the professional well‐being of the critical care nurse concerning the following categories: Structural architectural factors (in Related to work environment factors) and Nurse participation in hospital affairs (Related to work environment factors ‐Related to PES‐NWI factors).

## 5 Discussion

This systematic review illuminates the complex dynamics influencing the well‐being of critical care nurses, with an apparent imbalance favoring hindering factors overprotective ones, highlighting the profession's intrinsic challenges (Baraka et al., 2021–2023; D'Amico et al. 2022). Personal factors, notably resilience and effective coping skills, emerge as crucial buffers against the demanding nature of critical care nursing (Rhéaume and Breau 2022; McCormick et al., 2023). Mental and physical health are pivotal, with good sleep and physical exercise identified as key to maintaining well‐being (Vieira et al., 2022; Alzahrani et al., 2022).

The work environment significantly impacts nurses' well‐being, with organizational support, structural elements, and leadership being key determinants (Hall et al., 2022; Smith, Kokoczka, and Cottrell 2023). Notably, the Lavender Lounge concept represents an innovative structural‐architectural approach to supporting nurse well‐being (Smith, Kokoczka, and Cottrell 2023).

Within the PES‐NWI framework, supportive leadership, nurse participation in hospital affairs, and collegial nurse‐physician relationships are emphasized as critical to fostering a positive work culture (Rhéaume and Breau 2022; Bruyneel et al., 2023). However, the review identifies a gap in protective factors related to sociodemographic, socioeconomic, and other personal factors, suggesting areas for future focus and intervention.

## 6 Conclusions

The findings of this review reveal that critical care nurses’ well‐being is influenced by various factors. Protective elements, such as personal resilience and supportive work environments, play a crucial role in enhancing professional experience (McCormick et al., 2023; Smith, Kokoczka, and Cottrell 2023). Conversely, hindering factors, including sociodemographic challenges and the impact of the COVID‐19 pandemic, present significant obstacles (Jose, Dhandapani, and Cyriac, 2020; Heesakkers et al. 2021).

The review underscores the need for healthcare systems to address these challenges proactively. Improving protective factors and mitigating adverse ones is essential for safeguarding the well‐being of critical care nurses, vital for high‐quality patient care and effective healthcare systems (Bruyneel et al., 2021; Butera et al., 2021).

In conclusion, this review provides comprehensive insights into the factors affecting the well‐being of critical care nurses. It advocates for a holistic approach in healthcare management, focusing on enhancing both personal well‐being and professional support, to improve the overall quality of life and work satisfaction for these essential healthcare professionals.

### 6.1 Limitations

This study has several significant limitations that need to be acknowledged. First, it lacks the inclusion of qualitative studies, which could provide more nuanced insights into the personal experiences of nurses in critical care settings. The absence of these studies restricts our understanding of the complex, subjective aspects of nursing well‐being and job satisfaction.

Additionally, no meta‐analysis has been conducted due to the unavailability of relevant Randomized Controlled Trials (RCTs). This omission limits the strength and generalizability of our conclusions, as RCTs are often considered the gold standard for evaluating the effectiveness of interventions.

Another notable limitation is the variability in healthcare policies across different countries. The lack of standardized policies means that nurses’ experiences can vary widely depending on their geographical location and the specific health systems they work within. This diversity in experiences poses a challenge in drawing broad, universally applicable conclusions from the study.

Finally, a critical factor influencing our findings is the inclusion of nurses in the sample who were not specialized in critical care but were assigned to these units during emergencies, such as the COVID‐19 pandemic. This shift has likely hurt the well‐being of these nurses, given their lack of specialized training and preparation for the demands of critical care environments. The implications of this on our study's findings are substantial, as it introduces an additional variable that may skew our understanding of the well‐being of regularly stationed critical care nurses.

### 6.2 Implications for Nursing Management

Nursing management in critical care must focus on creating a nurturing work environment, balancing workloads, and ensuring adequate resources. Effective leadership, promoting open communication and teamwork, is key. Emphasizing personal health, mental wellness, and resilience programs is essential. Tailoring support to individual nurses’ needs and fostering professional development are also crucial. In response to challenges like the COVID‐19 pandemic, crisis management strategies and mental health support are vital. These comprehensive approaches aim to improve nurse well‐being, leading to better patient care and a robust healthcare system.

## Conflicts of Interest

The authors declare no conflicts of interest.

## Data Availability

The data that support the findings of this study are available from the corresponding author upon reasonable request.
